# Social representation and practices related to dementia in Hai District of Tanzania

**DOI:** 10.1186/1471-2458-14-260

**Published:** 2014-03-19

**Authors:** Declare Mushi, Amen Rongai, Stella-Maria Paddick, Catherine Dotchin, Chauka Mtuya, Richard Walker

**Affiliations:** 1 Kilimanjaro Christian Medical University College, Box 2240, Moshi, Tanzania; 2 Institute of Neuroscience, Newcastle University, Newcastle upon Tyne, UK; 3 Northumbria Healthcare NHS Foundation Trust, North Tyneside General Hospital, North Shields, UK; 4 Institute of Health and Society, Newcastle University, Newcastle upon Tyne, UK; 5 Institute for Ageing and Health, Newcastle University, Newcastle upon, Tyne, UK; 6 Partnership for Health and Development in Africa, Moshi, Tanzania

**Keywords:** Dementia, Social-cultural, Beliefs, Life experience

## Abstract

**Background:**

With the increasing number of people surviving into old age in Africa, dementia is becoming an important public health problem. Understanding the social dynamics of dementia in resource-poor settings is critical for developing effective interventions. We explored the socio-cultural beliefs surrounding dementia and the life experience of people with dementia (PWD) and their caregivers in the Hai District of Kilimanjaro, Tanzania.

**Methods:**

Cross-sectional qualitative design. Forty one PWD were purposively sampled from the Hai District of Kilimanjaro. Twenty five paired interviews with PWD and with caregivers, and 16 with caregivers alone, were conducted. Interviews were tape recorded, transcribed verbatim and analyzed using content analysis approach.

**Results:**

Forty one PWD (26 females), aged 70 years and older, were recruited but due to speech difficulties only 25 participated in the interviews. Married were 13, widow in 22 and widower 6. The majority, 33/41 were illiterate. PWD and carers perceived memory problems as a normal part of ageing. Dementia was commonly referred as "*ugonjwa wa uzeeni*" (disease of old people) or memory loss disease. The majority of PWD 13/12 and carers 7/16 did not know what dementia is or what causes it. Dementia was felt to be associated with stroke, high blood pressure, diabetes, old age, curse/witchcraft and life stress. Half of the participants had used modern care and alternative care such as herbs, prayers or traditional healers. Caregivers complained about the burden of caring for PWD and suggested that community organizations should be involved in addressing the problem.

**Conclusions:**

Knowledge about dementia is low and the symptoms are accepted as a problem of old age. PWD and carers demonstrate pluralistic behaviour in seeking help from modern care, prayers and traditional healers. The disease adds significant burden to family members. Family and caregivers need more education on early recognition of symptoms and cost effective management of dementia at family level. Faith-based organizations could play an important role in dementia interventions. At a national level effective policy and improvement of the health care system to address the needs of PWD and their families are imperative.

## Background

Despite escalating poverty and communicable diseases the number of people surviving into old age is increasing and statistics show that the number of people with dementia (PWD) is also increasing [1,2]. According to the WHO (2012) [3], worldwide, nearly 35.6 million people live with dementia. By 2030, this number is expected to double, and more than triple (115.4 million) by 2050, as populations continue to age. In 2009 the United Nations estimated that between 2009 and 2050 Africa would witness a 68% increase in people living to be 60 years or older. Statistics show that more than half (58%) of PWD live in low- and middle-income countries and an increase to more than 70% by 2050 has been projected. Similar estimates were also presented by Prince (2008).

In Africa, population aging is occurring at a faster rate [4,5]. The prevalence of PWD in developing countries is estimated to be 5% in those over the age of 65 years [2]. In 2003 in Tanzania, people aged above 60 years old were estimated to be 4% of the population and this figure is expected to increase to 10% by 2050 [6]. In 1998, the prevalence of PWD in Egypt was 5.9%. A recent review reported that the prevalence of dementia increase with age from 2% at 60 to nearly 13% at the age of 90 [4]. Trends show that the prevalence rate increases with age and more women than men are affected [4,7]. Although the fundamental cause of dementia is not known, available literature demonstrates the most important risk factors for dementia are stroke, increasing age, female gender, low education, low socio-economic status, cardiovascular disease and certain genetic markers [7]. Other risk factors include alcohol consumption and living in rural areas [8,9].

In sub-Saharan Africa (SSA), authors have found that stigma exists around neurological and psychiatric diseases. Family members hide, or do not report, dementia cases and this could be linked to lower prevalence rates reported from developing countries [10].

### Health care and social support

It has been well documented that health care systems and services in less developed countries do not have adequate capacity (human and technological) to address the increasing demand of chronic diseases [1]. Unlike western countries, where there are special programs for PWD such as memory clinics, geriatric services, social work and carer support programs, the majority of PWD in the developing world are poor and do not have access to even basic care. They are usually undiagnosed [11] and receive inadequate support from informal carers such as family members.

Caring for PWD is one of the burdens that the family or caregivers face. As part of cultural practice, older people in Africa are exempted or discouraged from carrying out complex functional tasks [11]. This practice (out of love and care) makes elders more dependent and might have consequences on cognitive function. On the other hand, due to rural–urban migration and the impact of HIV/AIDS on the younger population, elderly PWD have additional responsibility to care for themselves as well as grandchildren. The burden for women with dementia is even bigger because they have other domestic responsibilities.

Although chronic diseases such as dementia require long term care and treatment, there are no effective health policies for old age and the majority of old people do not have a pension and are not covered by any health insurance. Despite its socio- economic and health effects, dementia is not a public health priority in Tanzania. In Tanzania, for example, the 2003 policy for old people [6] admitted that the majority of old people live in poverty and uncertainty. Most studies have approached dementia from a biomedical perspective. In this study we adopted a social model [12] to explore the socio-cultural beliefs surrounding dementia and the life experience of PWD and their caregivers in the Hai District of Tanzania. In the current study dementia was defined as a syndrome caused by disease of the brain, usually of a chronic or progressive nature, in which there is disturbance of multiple higher cortical functions, including memory, thinking, orientation, comprehension, calculation, learning capacity, language and judgment [13].

The term ‘dementia’ is used to describe the symptoms that occur when the brain is affected by specific diseases and conditions. Symptoms of dementia include loss of memory, confusion and problems with speech and understanding [13].

## Methods

### Design and methodology

This was a cross-sectional community-based study which employed qualitative methods.

### Study area

Hai district is in east part of Kilimanjaro Region. The district has 14 health facilities including Machame District Referral hospital. Hai district has a population of 210,533 and the proportion of people aged 70 years and above is about 2% (Tanzania Census, 2012). However, the Hai demographic surveillance site (DSS) where the research was based had a population of 160,000 at the time of the 2009 census, on which the prevalence study was based, of whom 5% were aged 70 and over [11]. The majority of the population lives within 5 km from a health facility. The national referral hospital Kilimanjaro Christian Medical Center and Mawenzi Regional Referral Hospitals are just 25 km away. The population of Hai is mostly involved in both food and cash crop (coffee) production and animal keeping. Literacy level in the district is over 90%. Most families have access to a smallholding, and daily activities consist of agricultural work including cultivating and animal husbandry alongside running the household and taking care of family members.

#### Sampling procedures

The study was part of a larger study of the prevalence and treatment gap for dementia in Hai District. Hai district is an established demographic surveillance site (DSS). We purposively sampled 41 PWD identified by the prevalence study. For recruitment of PWD we considered their ability to express clearly, age 70 years and above, gender, village and marital status.

### Data collection methods

Two experienced social scientists (DM and AR) performed the interviews. In-depth interviews were performed in nine villages namely Mudio, Bomangombe, Mbweera, Nshara, Sawe, Kwasadala, Kware, Kyuu and Urori. Data collection took place between February and April 2011. Using Kleinman’s explanatory model data collection tools were developed to examine perception (causes and symptoms) of dementia, meaning attached to and experiences of PWD and their carers and their health seeking behaviors [12].

We conducted 25 paired interviews which involved both PWD and their carers and 16 interviews which involved carers alone. Paired interviewed was adapted during the data collection after noting that some of the PWD had difficulties in expressing themselves or remembering things. In this case, most of the information was given by carers. The majority of carers are family members who have lived with patients for more than 20 years. Interviews were conducted in Kiswahili at home and were recorded using a digital device. Each interview lasted for about 45 minutes. The key questions asked were

1. In your opinion what is dementia? (Probe how does the community describe it, specific names given to the disease, causes of dementia, symptoms of dementia)

2. Could you please tell me your experience with dementia/caring for a person with dementia? (Probe: Health seeking behavior, coping, types of treatment (modern, traditional, spiritual), Source of social support etc.

Ethical approval was obtained from the National Institute of Medical Research and locally from KCMU College Research and Ethics Committee. The objectives of the study were explained to study participants and written consent (where possible) was obtained from PWD and their carers in advance. Permission to record interviews was sought from study participants prior to commencing interviews. Confidentiality and anonymity were strictly observed.

### Data analysis

Recordings were transcribed verbatim and translated into English by three researchers (DM, CM and AR). Data were checked and cleaned and then stored as Microsoft Word files. Data were manually analyzed (independently by two researchers) using a content analysis approach [14] which included, identification of recurring themes, categorization and interpretation. Analysis was undertaken and discussed by the two social scientists involved in the study.

## Results

Forty one participants were purposively recruited. These included 25 PWD and 16 caregivers. Males were 15 and females 26. The age of patients was 70 and above with median age of 84 and 26 aged between 80 and 100. The majorities were widows 22, widower in 6 and married in 13. Nearly all patients (40) were from the Chagga ethnic group, while 35 were Christian and 6 were Muslim. All patients were farmers and 33 had no primary education. Seven had some primary education, while one was educated beyond primary. No one had a pension and only one patient had health insurance. A total of 16 caregivers (11 females and 5 males) participated in the study. Half were married, 5 were single and 3 were divorced. The age of caregivers ranged between 19 and 58 years old. Only 6 of the caregivers had formal employment. Most of the caregivers were the regular carers of PWD and had lived with patients for more than 10 years.

### Knowledge and perception of dementia

From our findings no participants knew the term dementia. Out of those interviewed 14 PWD and 21 carers saw the disease as un-common but referred to it as a normal disease of old people "*ugonjwa wa wazee*" (disease of old people), or "*ugonjwa wa kusahau*’ (memory loss disease/disease of forgetting). Across the interviews the phrase "*hii ni kawaida kwa wazee’* (meaning this is normal for old people) was mentioned several times by both patients and caregivers. Ten PWD and 17 caregivers could not describe the disease. The common response was "*I don’t know what the problem is*". During interviews, six PWD and 18 caregivers associated dementia with other diseases like high blood pressure, stroke, diabetes and malaria. Dementia was reported to be a result of old age, life stress, thinking deeply, death of dear ones (husbands, wife, son or daughter) in PWD and 9 caregivers. Four PWD and five caregivers related dementia to curses or witchcraft. Seven of PWD and six caregivers said they didn’t know the cause of disease. Most responded "*Mungu anajua*" (God knows). No participants mentioned that dementia could be inherited.

### Symptoms of dementia

Participants were asked to describe any symptoms of dementia which they knew. Their responses are summarized in the table below (Table 1).

**Table 1:** Summary of symptoms of dementia as reported by participants

**PWD**	**Caregivers**
"Memory loss"	"Forget the road or people, including their own children"
"Forget things or places"	"Forget to eat or to change clothes"
"Forgetting people"	"Lost in conversation"
"Repeating same stories"	"Talking stories of the past"
	"Difficult in keeping change during business- returning more or less change"
	"Sitting in one place for a very long time"
	"Doing/repeating the same things twice or more"
	"Shouting during the night"
	"Difficulty in eating"

In more than half of the interviews participants said that people in the community have little knowledge about this problem. When asked how and when they noticed the problem, about half of the caregivers said after a sudden fall (which was later recognized as a stroke to some of the patients) and others said it started slowly and the major sign was memory loss.

### Stigma against dementia

Although no strong stigma was attached to it, dementia is accepted as an old age problem and people believe nothing much can be done about it. One female caregiver said that

"*people do feel bad to have patients with dementia*".

Another respondent said

"*Majority of PWD do not have interaction. Family members feel shy to disclose their status*".

### Health seeking behaviour

Of 41 PWD, 22 had attended modern care, 13 had sought help from prayers in the church and 6 had been to traditional healers/herbalists. The majority 30 of interviewees reported pluralistic behavior in seeking care and had sought help from modern care and from prayers or traditional healers. We noted that participants were free to say that they had visited modern care, had spiritual help or seen the traditional healers.

Further probing revealed that caregivers reported that most of the time when they took patients for modern care it was due to other health conditions and not necessarily for dementia treatment. A 35 year old male carer said

"*Because we know there is no treatment for dementia in our area people take PWD to hospital for other treatment*".

When asked why they took PWD to alternative care, the common response was that

"*we think the problem is beyond human control*", "*Only God knows the solution*".

A female patient (80 years) complained that "*modern treatment is not helping and the situation is getting worse as days go on"*.

On the question of effectiveness of different forms of treatment, there were mixed feelings. The majority of PWD (14/25) and caregivers (10/16) who had been to seek modern care or prayers confessed that they didn’t see much improvement. However, 12/25 of PWD and 6/16 of caregivers had a positive attitude towards modern care. The common phrase was that "*the modern treatment is better because health workers are knowledgeable*". Seven caregivers believed that "*prayers are more effective*". A female patient aged 71 years old said "*Prayers are very helpful because you get encouragement*". No one commented positively on the effect of traditional healing. Almost all those who had been to traditional healers said they didn’t see an improvement. A female caregiver aged 47 years old said "*The only solution is to attend hospitals and to trust God*".

### Source of social support

Nearly all PWD receive social and economic support from close family members such as spouses, children, grand children, relatives or neighbors and only 8 caregivers said their PWD could manage their life without close supervision from caregivers. Majority of caregivers were females followed by grand children. As shown in Table 2, caring for PWD pose great challenge to caregivers because they can’t fully engage in other productive activities and sometimes it can cause conflicts.

**Table 2:** Impact of dementia and social support

**PWD**	**Caregivers**
*"I feel very bad but there is nothing I can do" (Male, 79 years)*	*"I can’t be productive because I have to be around all the time to watch over him" (Female,44 years)*
*"I am not happy, I can’t do my work I as I used to do" (Female, 75 year)*	*"They need close care otherwise they can go into a dangerous area*" *(Female aged 56)*
*"My family is treating me like a child, they don’t allow me to cook anymore" (Female, 71 years)*	*"Sometimes he is troublesome, so I have to talk slowly with him when administering medication" (Female, 26 years)*
	*"We are used to it and we make sure someone is around" (Male, 35 years)*
	*"Sometimes we have to lock them inside if we have other activities outside home" (Female, 20 years)*
	*"This is a burden because both of us we can’t engage in productive activities" (Male, 39 years)*

Participants were asked to what extent the disease has impacted on their life. Their responses are presented in Table 2:

### Views on how to address the problem

PWD and caregivers had expressed different views regarding what should be done to help PWD and their families

"*Doctor should educate us more because we don’t know much about this problem" (*Female carer, 34 years)

*"There should be someone or organizations to educate and help people at community level" (*Male carer, 44 years)

*"Most of PWD are poor so they should get support such as food, money and medical care*" (Male patient, 71 years)

*"Churches should open drop- in centers where take our patients during the day"* (Female carer, 28 years)

*"Churches and NGOs should actively be involved in this problem like in other social problems"* (Male carer, 36 years)

*"The government should look for proper medication for dementia" (*Male patient, 75 years old*)*

The quotations above shows that PWD have different needs which could be met by involving health care providers, NGOs, religious institutions and the government.

## Discussion

In this study we aimed to explore the socio-cultural beliefs surrounding dementia and the life experience of PWD and their caregivers in the Hai District of Tanzania. The study shed light on the facts that knowledge about dementia and its causes is low in this area and dementia is accepted as an old age problem. In addition PWD and their carers have pluralistic behavior in seeking care and dementia has a huge impact on the social and economic life of families of PWD. The findings from this study are generally similar to previous studies [3,15,16]. However, more insights on what issues to consider in designing community-based interventions in resource poor settings are discussed.

### Social demographic characteristics and social support

In this study, females with dementia constituted a larger proportion (63%). Nearly three quarters of PWD were single (widow/widower) meaning that they depend from family members for social support. The majority of PWD are poor and economically inactive which pose a great challenge to people who are demented and old. We further noted that all had no pension or health insurance meaning that they pay their health bills from out of pocket. PWD heavily depends from their children, grandchildren and close relatives for support. We also noted the interdependence between some of the PWD and their grandchildren. PWD provide support to their grandchildren and they also receive support and help from their grandchildren. However, challenges posed with this kind of interdependence in Tanzania context and particularly among elderly with cognitive difficulties need to be studied further.

### Knowledge: causes, symptoms, treatment and care

Knowledge of how people understand dementia and its impact is fundamental to designing clinical care and awareness raising programs. Knowledge about the causes of dementia was described using both a biomedical and a social model. The majority of the patients fell under a biomedical explanation as they associated dementia with diseases, old age and life experience (stress). Interestingly, no one mentioned that people could inherit dementia. Under the social model only a few participants associated dementia with curses or witchcraft. Recent studies have shown similar findings [17].

Nearly half of participants (both PWD and carers) could not describe the dementia. They kept; saying "*I don’t know what is the problem"* and that "*God knows*". In their context this means that it is the will of God. Normally chronic diseases, which people believe cannot be cured with medicine, are often seen as God’s will or punishment [18]. Connel et al. [19] commented that relating illness as God’s will reflect high value placed on religion in the African American Community. Such beliefs usually mean some degree of disease acceptance and that nothing can be done about it.

In line with other studies elsewhere, the majority of participants described and accepted dementia as a normal old age problem [3,19]. This may explain why most participants described the disease using a biomedical model. Although knowledge on symptoms was sufficient, knowledge of dementia causes and treatment was found to be inadequate. Only two medically confirmed risk factors (stroke & being old) were known to the study participants. Limited knowledge about dementia could be associated with health service factors and the nature of the disease. At health facility level the system doesn’t have special programs for PWD and maybe doctors, or health professionals, don’t explain the problem to the PWD or caregivers. With regard to the nature of disease, dementia is currently relatively rare (prevalence is 6.4%) [20] and this could explain why nearly a quarter (22%) of participants didn’t know about it. We also noted that, unlike other chronic diseases such as epilepsy and Parkinson’s disease [21], dementia was less associated with witch craft or curses and little stigma is attached to it in Hai [10,22].

### Health seeking behaviour

This study found that the majority of PWD seek health care in more than one health sector, including modern care, traditional sectors and prayer sessions. The biomedical sector seems to be favoured more when other options fail. Over half of PWD had visited modern care. However, it was not clear if they did so specifically for dementia or due to other health conditions. People have the perception that nothing can be done and so they also seek care from other sources, particularly if people relate the problem with supernatural powers. Religious services, such as counseling and prayer, are part and parcel of people’s life in Hai. A substantial number of PWD (30/41) had sought help from prayers in the church. This implies that if religious leaders are targeted and involved they could help in offering advice and support to community members. They could also offer a venue for training and a meeting point for PWD and their carers. Based on these findings, intervention for dementia requires effective coordination between modern care and informal health care.

### Experience and coping with dementia

As has been found elsewhere, PWD in Hai district suffer from multiple problems such as poverty, ageing challenges, other diseases, stigma, inadequate health care and lack of health insurance. PWD are cared for by informal, unpaid relatives or children who do not have knowledge and support skills [3]. Brodaty and Luscombe correctly argued that caregivers are at risk of becoming socially isolated because of the demanding nature of their role [23].

Older people in Africa are exempted, or discouraged from, carrying out complex functional tasks. This makes elders more dependent and might have consequences for their cognitive function [11].

Although people believe nothing can be done about dementia, participants expressed views about the burden of care for PWD and the need for improved care and support of PWD and their families. Participants expressed that support such as food, money, appropriate information and care centres could help reduce the burden of care. Providing support to carers on the caring process and development of relevant skills should be the focus of any intervention at a community level. Interventions, such as support groups, have proved to be effective in reducing the burden on the family [24].

In the study area there is no governmental organization or programme addressing the problem of dementia. Unlike in the west, where there are special programs for PWD, such as memory clinics and Cognitive Stimulation Therapy (CST). Previous studies in the Hai district have reported that there are no health facilities which meet the needs of people with cognitive impairment [22,25]. Participants had the view that churches could play an important role in this area through the establishment of day centres for older people in churches, where relatives could leave their PWD. This could allow family members or other caregivers to engage in more productive work [26].

It is worth noting that in the Hai district, churches are very active in health care provision and in each parish there are two committees (health committee and committee for the needy) which deal with various social problems in the community regardless of peoples’ religious background. In designing community based interventions for people with dementia planners could exploit their potential.

According to WHO (2012) [3] only eight countries worldwide currently have national programs in place to address dementia. A recent report by WHO and Alzheimer’s Disease International [3] recommends that programs should focus on improving early diagnosis, raising public awareness about the disease and reducing stigma, and providing better care and more support to caregivers. The same report recommends that caregivers should be involved in designing programs to provide better support for people with dementia and those looking after them. Moreover, in resource limited countries like Tanzania, deliberate efforts are needed at a national level as the elderly population is expected to increase by more than 10% by 2050 [6]. WHO promote preventive programs, both in high and low income countries, because they could delay the need for people to enter high-cost care [3].

### Study limitation

This study had two major limitations. First, biases might have been introduced by individuals who might have given socially desirable answers particularly when probing about the challenges facing carers. Secondly, the fact that we conducted paired interviews, the views of care givers might have been more dominant in some aspects than those of PWD.

## Conclusion

This study sheds light on the fact that knowledge about dementia and its causes in this area of Tanzania is in adequate and it is generally accepted as an old age problem. PWD and their carers have pluralistic behaviour in seeking care from modern care, prayers and traditional healers. Since dementia is considered to be part of normal ageing, and since there is little or no awareness of risk factors for dementia, medical intervention is rarely sought. Based on our findings, intervention for dementia requires effective coordination between modern care and informal health care. Despite the social and economic impact of dementia, the majority of the carers and family members lack basic skills on how to care for people with dementia. Family and caregivers need more education on causes, early recognition of symptoms and cost effective management of dementia at a family level. Faith-based organizations could play an important role in community-based dementia interventions. At national level effective policy, and improvement of the health care system to address the needs of PWD and their families, are imperative. More research should be carried to develop culturally appropriate family based interventions to support PWD and their caregivers in poor resource settings.

## Competing interests

The authors declare that they have no competing interests.

## Authors’ contributions

DM. designed the study, collected and analyzed data and prepared the manuscript for publication. AR and CM completed data collection and conducted initial data analysis. SMP was involved in the identification of patients and in preparation of the article. RW and CD provided scientific advice and input during study design and writing up. All authors read and approved the final paper.

## Pre-publication history

The pre-publication history for this paper can be accessed here:

http://www.biomedcentral.com/1471-2458/14/260/prepub
